# CpG methylation changes associated with hyperglycemia in type 1 diabetes occur at angiogenic glomerular and retinal gene loci

**DOI:** 10.1038/s41598-024-82698-9

**Published:** 2025-05-08

**Authors:** Xiaojian Shao, Sophie Le Fur, Warren Cheung, Marie-Pierre Belot, Kevin Perge, Natacha Bouhours-Nouet, Candace Bensignor, Lucie Levaillant, Bing Ge, Tony Kwan, Mark Lathrop, Tomi Pastinen, Pierre Bougnères

**Affiliations:** 1 Digital Technologies Research Center, National Research Council Canada, Ottawa, ON K1A 0R6 Canada; 2 Department of Biochemistry, Microbiology and Immunology, University of Ottawa, Ottawa, ON K1H 8M5 Canada; 3 Groupe d’Études Diabète-Obésité-Croissance, Assoc1901, Chaville, 92370 France; 4 Genomic Medicine Center, Children’s Mercy - Kansas City and Children’s Mercy Research Institute, Kansas City, MO 64108 USA; 5 Endocrinologie Pédiatrique, Hôpital Mère Enfant, 69677 Lyon, Bron, France; 6 Endocrinologie et diabétologie pédiatriques , Hôpital universitaire, Angers Cedex 9, 49933 France; 7 CHU Dijon Bourgogne, Hôpital d’Enfants, Dijon, 21000 France; 8 Department of Human Genetics, McGill University and McGill Genome Center, Montreal, QC H3A 0G1 Canada

**Keywords:** CpG methylation, Hyperglycemia, Type 1 diabetes complications, Angiogenesis, DNA methylation, Type 1 diabetes, Diabetes complications

## Abstract

Chronic hyperglycemia is a major risk factor for glomerular or retinal microangiopathy and cardiovascular complications of type 1 diabetes (T1D). At the interface of genetics and environment, dynamic epigenetic changes associated with hyperglycemia may unravel some of the mechanisms contributing to these T1D complications. In this study, blood samples were collected from 112 young patients at T1D diagnosis and 3 years later in average. Whole genome-wide bisulfite sequencing was used to measure blood DNA methylation changes of about 28 million CpGs at single base resolution over this time. Chronic hyperglycemia was estimated every 3–4 months by HbA1c measurement. Linear regressions with adjustment to age, sex, treatment duration, blood proportions and batch effects were employed to characterize the relationships between the dynamic changes of DNA methylation and average HbA1c levels. We identified that longitudinal DNA methylation changes at 815 CpGs (with suggestive p-value threshold of 1e-4) were associated with average HbA1c. Most of them (> 98%) were located outside of the promoter regions and were enriched in CpG island shores and multiple immune cell type specific accessible chromatin regions. Among the 36 more strongly associated loci (p-value < 5e-6), 16 were harbouring genes or non-coding sequences involved in angiogenesis regulation, glomerular and retinal vascularization or development, or coronary disease. Our findings support the identification of new genomic sites where CpG methylation associated with hyperglycemia may contribute to long-term complications of T1D, shedding light on potential mechanisms for further exploration.

**Supplementary Information:**

The online version contains supplementary material available at 10.1038/s41598-024-82698-9.

## Introduction

Both microvascular (retinopathy, nephropathy, and neuropathy) and cardiovascular disease occur decades after T1D onset^1^, resulting in high morbidity and mortality. While degree and duration of hyperglycemia are the main drivers of these complications^2^, the molecular mechanisms of how excess glucose leads to specific organ damage are only partially known. The causes of T1D micro- and macroangiopathy are multifactorial and involve genetic susceptibility, epigenetic alterations induced by the disturbed metabolic status, dyslipidemia, elevated blood pressure, insulin resistance, advanced glycation end products, increase in local growth factors, oxidative stress^3^ and added factors such as smoking or overweight.

Poor glycemic control is the major determinant of the risk and onset timing of micro or macrovascular complications. It is clinically actionable since the genesis and progression of these complications of T1D are significantly limited by an improved glycemic control. However, improving glycemic control does not reverse established complications or, more surprisingly, does not seem to mitigate the deleterious consequences of earlier exposure to poor glycemic control, an effect termed “metabolic memory”^4^. Although the concept of metabolic memory is debated^5^, it seems that early exposure to high blood glucose, even followed by years of better glycemic control, has durable effects on T1D complications^4,6^.

Epigenetic marks are suspected to offer mechanisms for this lasting effect of hyperglycemia^3,7–10^. Indeed, DNA methylation is a major epigenetic modification and its alterations can lead to dysregulated gene transcription and cell dysfunction in specific tissues. Since DNA methylation is both persistent in post-mitotic cells and inherited during cell division, its durable changes might prolong the effects of previous glycemic history^7,11–13^.

Studies investigating DNA methylation in T1D patients have identified specific differentially methylated regions (DMRs) linked to long-term complications^14–18^, glycemic control^19,20^ or metabolic memory^21^. These studies will be detailed in the Discussion section. Exploring the relationship between DNA methylation and hyperglycemia in T1D patients could uncover critical molecular alterations associated with glycemic control and shed light on risk evaluation or prevention approaches. To explore DNA methylation in T1D patients in their early post diagnostic years, we adopted a longitudinal follow up study design to avoid the paramount difficulty of inter-individual methylation variation. In contrast to previously used array-based technologies which bias towards promoter and CpG island regions, the whole genome-wide bisulfite sequencing (WGBS) measures approximately 28 million CpGs across promoters, enhancers and other intergenic regions.

## Methods

### Study design and cohort - sample collection

We recruited 112 children from the Isis-Diab cohort^22^ according to autoimmune T1D classic criteria^23^. All of these patients are of European ancestry. Patients’ parents were provided with comprehensive information about the investigational nature of the study and subsequently signed their informed consent which was agreed by Comité de Protection des Personnes (CPP) under the reference number DC-2008-693; NI 2620.

Blood samples were extracted during the regular medical assessment of patients as part of their clinical management. Hyperglycemia was estimated by the mean HbA1c (glycated hemoglobin) level measured every 3–4 months during the period elapsed between the two blood samples used to measure methylation, the first one (baseline) taken within a 1–3 days of insulin treatment onset, the other 1 to 5 years later.

### Isolation of genomic DNA

Peripheral blood mononuclear cells (PBMC) were isolated from fresh blood using a density gradient. Five milliliters of fresh blood were mixed with 5 ml of NaCl 154 mM, and 5 ml of Lymphoprep solution (Eurobio, Paris, France) was added to the diluted blood and centrifuged for 20 min at room temperature at 800 g. After centrifugation, the interphase containing PBMC was carefully aspirated and the cells were mixed with NaCl. The cell suspension was centrifuged at 300 g and the cell pellet washed with PBS. PBMC were frozen at -80 °C. Nucleic acids were extracted from PBMC using Gentra Puregene blood kit (Qiagen, Hilden, Germany).

### WGBS DNA methylation protocol

WGBS gDNA library preparations were carried out using the Lucigen NxSeq^®^AmpFREE Low DNA Library Kit (Lucigen) with an added bisulfite conversion step. Briefly, 1–2 µg of gDNA was fragmented to 300–350 bp peak size using the focused-ultrasonicator E210 (Covaris, Woburn, MA, USA) to generate double-stranded DNA with 3’ or 5’ overhangs. Fragment size distribution was controlled on a Bioanalyzer DNA 1000 Chip (Agilent, Mississauga, ON, Canada). End repair, sample purification with AMPure beads (Beckman Coulter, Mississauga, ON, Canada), adenylation of 3’ ends, and adaptor ligation was carried out as per Illumina’s recommendations. The ligation product was cleaned-up by one AMPure purification step, the purified DNA was then analyzed on a Bioanalyzer High Sensitivity DNA Chip (Agilent) and quantified by PicoGreen before undergoing bisulfite conversion using the Zymo EZ-96 DNA Methylation-Gold MagPrep kit (Zymo Research) according to manufacturer’s protocol. Bisulfite-converted DNA was quantified using OliGreen (Life Technologies) and based on quantity amplified by four to six cycles of PCR using the Hifi Uracil + DNA polymerase (Kapa Biosystems, Woburn, MA, USA) according to manufacturer’s protocol. Amplified libraries were validated and quantified on Bioanalyzer High Sensitivity DNA Chips and underwent 150 bp paired-end sequencing on Illumina HiSeqX at the McGill Genome Centre.

### WGBS data process

The generated FASTQ raw reads were processed at either McGill Genome Centre or Genomic Medicine Center of Children’s Mercy Hospital. For the batch of the samples processed at McGill genome center, the GenPipes pipeline was adopted with the methylseq mode^24^. Specifically, the WGBS paired-end raw reads were trimmed for quality (phred33 ≥ 30) and Illumina adapters using Trimmomatic (version 0.36)^25^. The trimmed reads were aligned to the bisulfite-converted hg19/GRCh37 reference genome using Bismark (version 0.18.2)^26^ with Bowtie 2 (version 2.3.1)^27^ in paired-end mode with default settings.

For the WGBS fastq reads processed at Genomic Medicine Center of Children’s Mercy Hospital, the Epigenome Pipeline available on the DRAGEN Bio-IT platform (Edico Genomics/Illumina) was utilized. Initially, the WGBS paired-end raw reads were de-multiplexed into FASTQ files using Illumina’s bcl2Fastq2-2.19.1 software. Subsequently, the de-multiplexed reads were quality trimmed (phred33 ≥ 20) and the Illumina adapters were removed using trimgalore v.0.4.2 (https://www.bioinformatics.babraham.ac.uk/projects/trim_galore/), which is a wrapper tool incorporating Cutadapt^28^ and FastQC (https://www.bioinformatics.babraham.ac.uk/projects/fastqc/). The alignment on the trimmed reads was accomplished using DRAGEN EP v2.6.3 or a later version in paired-end mode, following the directional methylation protocol presets. Alignments were calculated for both strands, and the unique alignment with the highest quality was retained.

The aligned BAM files from both pipelines were de-duplicated using Picard (version 2.9)^29^. Methylation states were then called for each cytosine in the CpG, CHG, and CHH contexts using bismark or DRAGEN. The DNA methylation level of each CpG was determined by calculating the ratio of methylated reads to the total number of sequenced reads. CpG sites located within sex chromosomes, overlapping with SNPs (dbSNP 137), the DAC Blacklisted Regions^30^, or Duke Excluded Regions (generated by the ENCODE project) were excluded. Additionally, CpG sites with less than 15X read coverage were discarded.

### Statistical analysis

To infer the longitudinal DNA methylation level changes between two time points for each CpG, the proportion testing was applied where only CpGs with > = 30X for both time points were retained and nominal p-value < 0.01 as significant. To investigate the association between changes in DNA methylation level (delta beta) and the average HbA1c during the observed period, a linear regression model (LM) was constructed and applied to CpGs covered by at least 30 individuals with read coverage > = 15 for both time point measures. The model accounted for confounding factors such as age onset, sex, T1D duration, and changes of estimated blood cell proportions, as well as sequencing batches. The R function lm() was utilized to fit the model, and p-values were calculated for the variables of interest.

Considering the limited sample size, a nominal p-value threshold of < 1e-4 was employed to determine HbA1c-associated CpGs (hDMCs), acknowledging that this represents a suggestive rather than statistically significant threshold. Blood deconvolution was performed using constrained linear projection^31^ through the projectMix function of the RefFreeEWAS package. A custom panel of 30,455 CpGs specific to cell types (Neutrophil, Monocyte, B-cell, and T-cell) with hypomethylated and hypermethylated patterns was employed for blood reference epigenome profiles.

### Genome features and function enrichment analysis

To obtain genome feature annotations, we retrieved tables from the UCSC genome browser (https://genome.ucsc.edu/) based on the hg19 build version. The downloaded annotation tables included various genomic elements such as transcription start sites (TSSs), 3’UTRs, 5’UTRs, first exons, exons, introns, and transcription end sites (TESs). For promoter regions, we considered both TSS200 (200 bp from TSSs) and TSS1500 (1500 bp from TSSs). Additionally, we obtained the CpG islands (CGI) annotation table from the UCSC genome browser. Furthermore, we defined CGI north and south shores as the 2-kb flanking sequences upstream and downstream of CGIs, respectively. The north and south shelves were defined as the 2-kb flanking sequences beyond the shores.

To assess the enrichment of genomic features in relation to hyperglycemia exposure differentially methylated CpGs (DMCs), we conducted genome feature enrichment analyses using Fisher’s exact test. The background set for significance testing comprised all testable CpGs which were used in the linear regression analyses. For gene ontology enrichment analyses, we employed the Homer tool^32^ (version 4.11) where hDMCs were annotated to their closest genes based on the distance to the TSS or the genes they are located within. Terms were identified as significant if the P-value < 0.05.

To investigate gene function for the methylation changes more closely associated with HbA1c, we screened PubMed by entering “gene name” with the following keywords “angiogenesis”, “vascularization”, “kidney”, diabetic nephropathy”, glomerulus”, “retina”, “coronary”.

## Results

### Descriptions of the T1D cohort

For the longitudinal DNA methylation change analysis upon hyperglycemia, DNA methylation profiles were measured at two time points of 112 T1D patients (pairs). The main characteristics of these 112 T1D patients are illustrated in Table 1. Sixty-four males and 48 females were aged 1.6 to 15.3 years at time of T1D diagnosis. At time of the second blood sampling, T1D duration averaged 3 years (range 1.2–5.1 years). Mean HbA1c level measured every 3–4 months during this period was 7.7%, with standard deviations (SD) of 0.9% and a distribution of values shown in Figure S1 (Additional file 1). Blood proportions were estimated from the DNA methylation profiles and the changes of blood proportions between the two time points were calculated.

**Table 1:** Main characteristics of the studied participants and blood cell proportions (Mean ± SD).

	All	Male	Female
N	112	64	48
Age at diagnosis (yr)	9.7 ± 3.7	10.0 ± 3.8	9.4 ± 3.7
T1D duration (yr)	3.0 ± 1.1	3.0 ± 1.0	3.1 ± 1.1
HbA1c (%)	7.7 ± 0.9	7.7 ± 1.1	7.6 ± 0.6
B cell proportion changes (%)	0.12 ± 1.1	0.03 ± 1.11	0.24 ± 1.08
T cell proportion changes (%)	0.06 ± 0.80	0.001 ± 0.81	0.13 ± 0.78
Monocyte proportion changes (%)	0.21 ± 1.34	0.06 ± 1.34	0.40 ± 1.33
Neutrophil proportion changes (%)	0.12 ± 1.16	-0.002 ± 1.23	0.29 ± 1.05
Megakaryocyte proportion changes (%)	0.14 ± 0.79	0.20 ± 0.83	0.05 ± 0.74

### Pairwise longitudinal DNA methylation changes using proportion testing results

The average read coverage of CpGs along the whole genome for the 112 pairs (i.e., 224 samples) was 15-fold (Additional file 2: Table S1, and Additional file 1: Figure S2A). After removing CpGs with less than 15X, located at blacklist regions as well as sex chromosomes, close to 18.3 million CpGs with at least 10 longitudinal pairs with each CpG at > = 15X were remained for downstream analysis. We observed that the paired longitudinal measurements/samples for these 112 children were well clustered according to their methylation profiles (Additional file 1: Figure S2B).

**Fig. 1:**
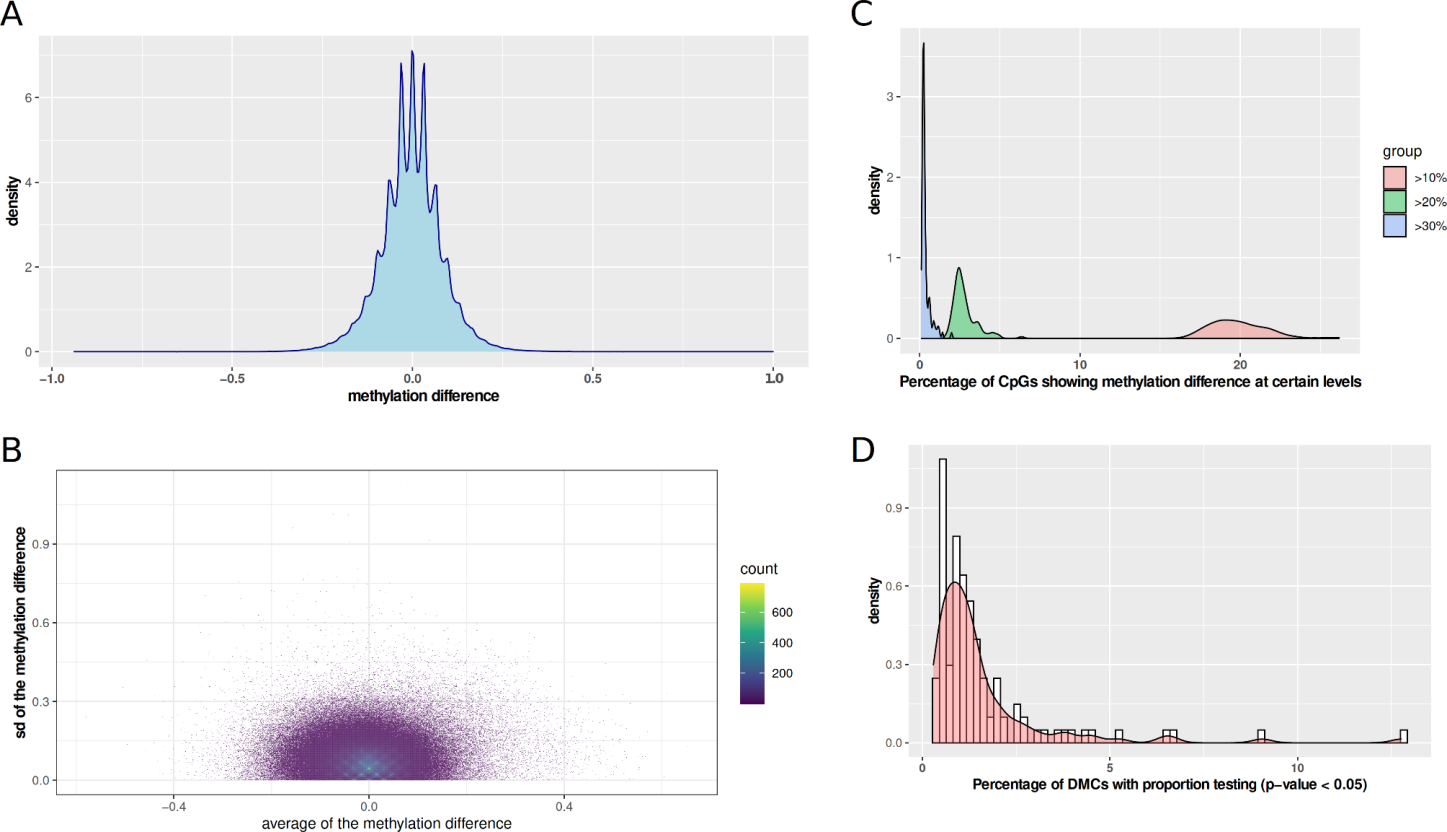
Characterization of DNA methylation changes upon exposure to hyperglycemia (average HbA1c). (**A**) Distribution of the DNA methylation changes. (**B**) Scatter plot between the average DNA methylation difference and standard deviation of DNA methylation changes over all CpGs. The heatmap colors depicted in the legend were used to visually represent the density of CpGs. (**C**) Distribution of the percentage of CpGs exhibiting varying levels of differential methylation changes (> 10%, > 20% and > 30%) across samples. (**D**) Distribution of the percentage of CpGs showing significant methylation difference using proportion testing (p-value < 0.01) per sample.

We initiated the comparison of the DNA methylation difference for each CpG within individual pairs. To avoid the influences of low read coverage on calculating the methylation difference, we required CpGs with ≥ 30X for both time-point measurements for this analysis (*n* = 2,891,038 with at least one pair). Most of the CpGs displayed small mean absolute methylation difference (e.g., approximately only 10% of these CpGs exhibited mean methylation differences of 12% or more) and had low standard deviation (Fig. 1A, B). When considering each of the measured CpG pairs at ≥ 30X across 112 children (*n* = 8,897,316), the mean absolute value of the methylation changes between the two time points is 6.5 ± 5.8% (SD). There was no difference between male and female groups (Additional file 1: Figure S3). Besides three peaks in the middle, including the large peak close to zero and two other peaks with minor methylation changes (e.g., ~±3% changes), another two peaks at DNA methylation changes of ~ 6.5% were observed. We considered the CpGs enriched in the latter peaks as reflecting the epigenetic response to the exposure to the diabetic state, including chronic hyperglycemia. By requiring ≥ 10% methylation difference between the two time points, only 15,540 CpGs demonstrated either ≥ 10 hypo-methylated samples or ≥ 10 hyper-methylated samples. Among those CpGs, 5,061 CpGs showed exhibited divergent response to the exposure to the diabetic state (i.e., ≥ 10 hypo-methylated samples and ≥ 10 hyper-methylated samples). Moreover, these numbers decreased to 172 and 65 when a ≥ 20% methylation change was required. When evaluating the number of CpGs showing methylation changes upon exposure to the diabetic state, we observed that roughly 19.86%, 2.79% and 0.36% of the CpGs showed methylation differences > 10%, 20% and 30%, respectively, when comparing the two time point samples (Fig. 1C). These proportions of longitudinal DMCs per sample were not correlated with other phenotypes including age, average HbA1c, or T1D duration.

We further applied proportion test to statistically infer the dynamic changes of DNA methylation level of CpGs with ≥ 30X (Fig. 1D). The proportion of longitudinal DMCs (p-value < 0.01 at proportion testing) per sample was not correlated with the average HbA1c level (correlation *R* = 0.05, p-value = 0.57). Moreover, a set of CpGs showed longitudinal DMCs across multiple individuals. For instance, 660 and 193 CpGs showed longitudinal methylation changes over time on more than 5 and 10 individuals, respectively. This indicated that the diabetic state might have a site-specific influence, rather than a global influence on DNA methylation changes.

### Differentially methylated CpGs associated with exposure to hyperglycemia

To identify differentially methylated CpGs associated with exposure to hyperglycemia (hDMCs), we regressed the difference in DNA methylation values between baseline and follow-up PBMC samples against the average HbA1c level. The model was further adjusted to age at first sampling, T1D duration, differences in blood cell proportions between the two time-points, and batch effects.

**Fig. 2:**
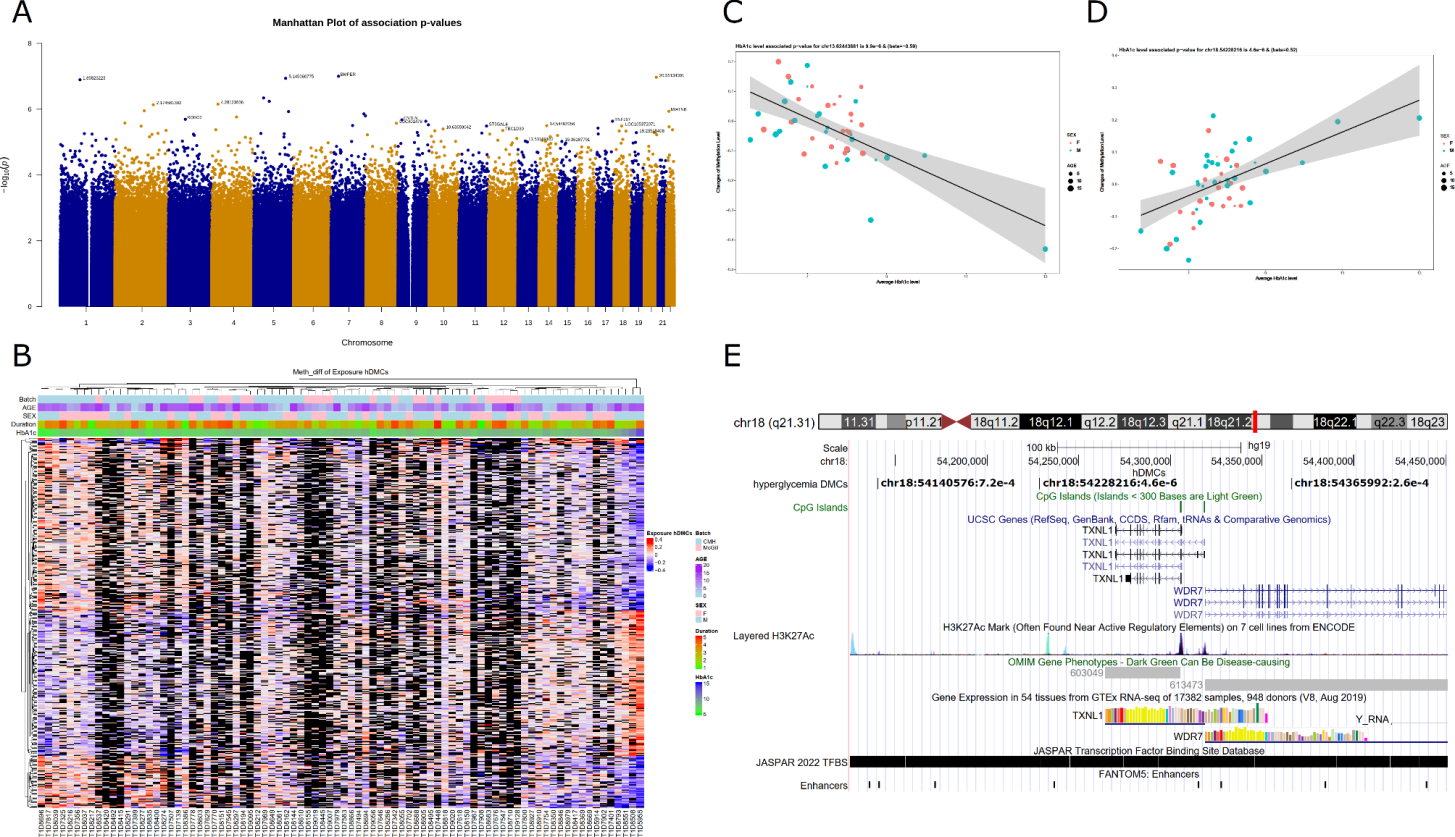
The distribution of differentially methylated CpGs associated with average HbA1c. (**A**) Manhattan plot of p-values from the average HbA1c association analysis. The top hDMCs with p-value < 1e-5 per chromosome were labelled, with associated genes or CpG coordinates displayed based on whether the hDMCs were located at gene region or in intergenic regions. (**B**) Heatmap of HbA1c associated hDMCs at p-value < 1e − 4. Different phenotype features (including different sequencing platforms, sex, age onset, T1D duration, average HbA1c) are illustrated in the top plots. Missing data are indicated with black color. (**C**) Scatter plot for hDMCs negatively correlated with average HbA1c level. (**D**) Scatter plot for hDMCs positively correlated with average HbA1c. Sex and age are indicated with different colors and different dot size of dots. (**E**) UCSC track browser for the identified significant CpG in (D). This CpG is located upstream of WDR7 and downstream of *TXNL1*. The hyperglycemia DMCs track indicates hDMCs with p-value < 1e-3 where chromosome id, CpG position and hDMC association p-values were listed.

Due to the limited sample size, we did not observe any significant DMCs after multiple test corrections at an FDR of 0.05 for both models. However, when using a nominal p-value < 1e-3 and a more stringent threshold of p-value < 1e-4, we observed a total of 8375 and 815 hDMCs, respectively. Figure 2A shows the genome-wide distribution of the top hDMCs using a Manhattan plot. Among these, 5078, 482 hDMCs showed negative correlations between DNA methylation changes and the average HbA1c level, while 3297, 333 hDMCs showed positive correlations, respectively. Additionally, the volcano plot showing positive and negative correlated hDMCs was presented in Additional file 1: Figure S4. The pattern of DNA methylation changes of the hDMCs associated with HbA1c at p-value < 1e-4 is illustrated in Fig. 2B. Three major CpG modules showing positive and negative correlations were observed. Table 2 presents the 36 significant hDMCs associated with average HbA1c level (p-value < 5e-6) with the top examples shown in Fig. 2C-E. For instance, one of the interesting hDMC is located at upstream of *WDR7* and downstream of *TXNL1* as indicated in Fig. 2E. The full list of the 815 associated hDMCs is shown in Additional file 3: Table S2.

**Table 2:** The 36 hDMCs list with p-value < 5e − 6 for association with average HbA1c level.

chr.position	*P*-value	Beta	Annotation	Distance_to_TSS	Gene_Name	Gene_Type	Gene_Description
chr7.34125622	9.99E-08	-0.4723	exon (NM_133468, exon 14 of 16)	180,472	BMPER	protein-coding	BMP binding endothelial regulator
chr20.55134301	1.07E-07	-0.5767	Intergenic	-18,317	LINC01716	ncRNA	long intergenic non-protein coding RNA 1716
chr5.145060775	1.16E-07	-0.5301	Intergenic	154,129	PRELID2	protein-coding	PRELI domain containing 2
chr1.89823223	1.28E-07	-0.6859	Intergenic	-6212	GBP6	protein-coding	guanylate binding protein family member 6
chr5.44588593	4.56E-07	-0.7885	Intergenic	-78,210	LINC02224	ncRNA	long intergenic non-protein coding RNA 2224
chr5.70526460	5.77E-07	-0.3183	intron (NR_033968, intron 1 of 3)0.2	28,661	GUSBP9	pseudo	GUSB pseudogene 9
chr4.28123636	7.03E-07	-0.8578	Intergenic	-697,567	MIR4275	ncRNA	microRNA 4275
chr2.174581392	7.32E-07	-1.0757	Intergenic	247,215	SP3	protein-coding	Sp3 transcription factor
chr2.133312714	1.12E-06	-0.7042	intron (NM_001508, intron 1 of 1)	115,063	LYPD1	protein-coding	LY6/PLAUR domain containing 1
chr22.27102211	1.16E-06	0.3978	intron (NR_110543, intron 1 of 5)	33,406	MIATNB	ncRNA	MIAT neighbor
chr5.158355293	1.18E-06	-0.5056	intron (NM_182708, intron 5 of 14)	169,670	EBF1	protein-coding	EBF transcription factor 1
chr7.149569157	1.39E-06	1.1053	promoter-TSS (NM_001367793)	-895	ATP6V0E2	protein-coding	ATPase H + transporting V0 subunit e2
chr7.156711203	1.59E-06	0.371	Intergenic	-25,302	LMBR1	protein-coding	limb development membrane protein 1
chr4.112542915	1.72E-06	0.639	Intergenic	-523,694	FAM241A	protein-coding	family with sequence similarity 241 member A
chr3.77156286	2.03E-06	-0.7995	intron (NM_001128929, intron 1 of 24)	9124	ROBO2	protein-coding	roundabout guidance receptor 2
chr9.17153943	2.12E-06	0.3559	intron (NM_001365029, intron 2 of 25)	18,906	CNTLN	protein-coding	centlein
chr9.126085507	2.33E-06	-0.2832	Intergenic	-32,968	CRB2	protein-coding	crumbs cell polarity complex component 2
chr17.74228423	2.33E-06	0.335	intron (NM_052916, intron 1 of 18)	8150	RNF157	protein-coding	ring finger protein 157
chr2.45225960	2.64E-06	-0.4213	Intergenic	10,630	SIX2	protein-coding	SIX homeobox 2
chr8.138829527	2.68E-06	0.684	intron (NR_161374, intron 7 of 7)	266,260	LOC401478	ncRNA	uncharacterized LOC401478
chr9.138279766	3.00E-06	0.4447	Intergenic	44,672	C9orf62	protein-coding	chromosome 9 open reading frame 62
chr7.98062878	3.14E-06	-0.3141	Intergenic	-32,477	BAIAP2L1	protein-coding	BAI1 associated protein 2 like 1
chr14.54492056	3.18E-06	-0.4196	Intergenic	-68,448	BMP4	protein-coding	bone morphogenetic protein 4
chr18.34261812	3.24E-06	0.3796	intron (NM_001281739, intron 12 of 23)	-18,432	LOC105372071	ncRNA	uncharacterized LOC105372071
chr11.126307571	3.25E-06	0.6541	intron (NM_001301097, intron 11 of 15)	31,590	ST3GAL4	protein-coding	ST3 beta-galactoside alpha-2,3-sialyltransferase 4
chr2.118808780	3.28E-06	-0.33	Intergenic	-37,135	CCDC93	protein-coding	coiled-coil domain containing 93
chr22.27946359	3.41E-06	1.0319	Intergenic	239,748	LINC02554	ncRNA	long intergenic non-protein coding RNA 2554
chr17.8058934	3.76E-06	1.2495	Intergenic	-3213	PER1	protein-coding	period circadian regulator 1
chr10.63659042	3.99E-06	-0.1884	Intergenic	-2415	ARID5B	protein-coding	AT-rich interaction domain 5B
chr4.17982399	4.22E-06	0.6695	intron (NR_158566, intron 1 of 5)	40,978	LCORL	protein-coding	ligand dependent nuclear receptor corepressor like
chr22.43790250	4.23E-06	0.5126	Intergenic	-6086	LINC01639	ncRNA	long intergenic non-protein coding RNA 1639
chr2.232597736	4.43E-06	-0.3504	exon (NM_001291018, exon 4 of 4)	19,713	MIR1244-3	ncRNA	microRNA 1244-3
chr12.65220378	4.44E-06	0.2587	intron (NM_001330186, intron 1 of 11)	1952	TBC1D30	protein-coding	TBC1 domain family member 30
chr10.26142266	4.55E-06	-0.9543	Intergenic	80,372	LOC101929073	ncRNA	uncharacterized LOC101929073
chr18.23373363	4.60E-06	0.2854	Intergenic	297,213	SS18	protein-coding	SS18 subunit of BAF chromatin remodeling complex
chr18.54228216	4.62E-06	0.5167	Intergenic	77,606	TXNL1	protein-coding	thioredoxin like 1

The hDMCs associated with average HbA1c were sorted by p-value. CpG chromosome and position, regression p value, beta value (coefficient) and the annotated closest gene information (including genomic Annotation, Distance to TSS, Gene Name, Gene Type, and Gene Description of the closest gene) using Homer are provided.

### Genome feature and function enrichment analysis of hyperglycemia exposure DMCs

**Fig. 3:**
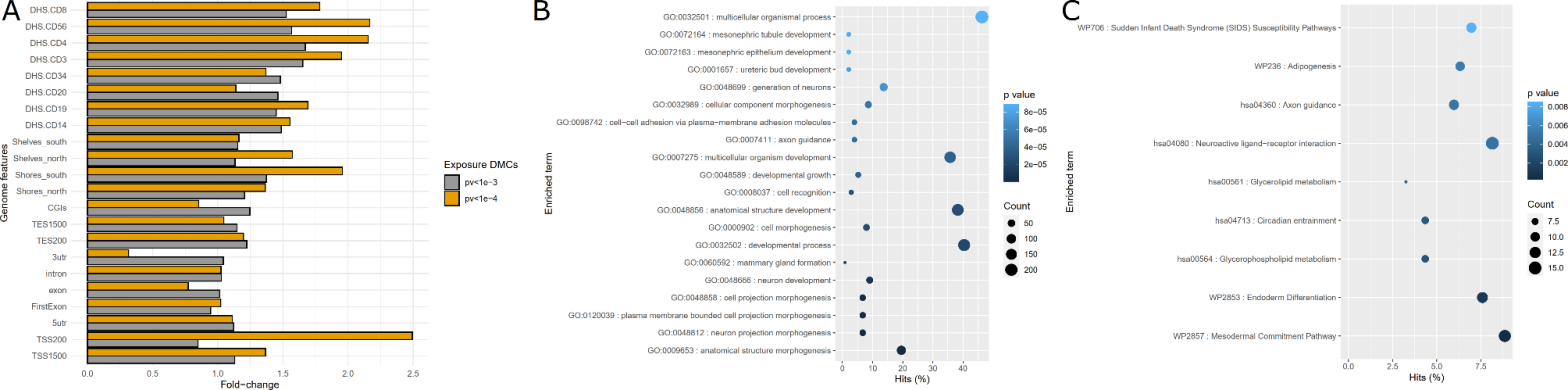
Genome feature and functional enrichment analysis of hDMCs. (**A**) Genome feature enrichment results. (**B**) GO BP enrichment results. (**C**) Wikipathway and KEGG pathway enrichment results. For (**B**) and (**C**), x-axis represents the percentage of the hDMC genes in the term. Dot size represents the number of hDMCs associated genes in the specific term. The enriched items were ordered by p-values.

We first performed the genomic feature enrichment analysis for the hDMCs. Only a very small number of DMCs were located at promoter regions and these hDMCs were mainly enriched at CpG island shores, Th1 and Th2 cell type specific DNase I hypersensitive sites (DHS) regions as well as CD3, CD4 and CD56 cell type specific DHS regions (Fig. 3A).

We then searched for functional enrichment analysis for the genes associated with DMCs associated with average HbA1c. For the 815 hDMCs (p-value < 1e-4) associated genes (*n* = 775), we analyzed the enrichment using Homer (annotatepeaks function)^32^. Interestingly, these genes were enriched in the gene ontology (GO) biological process (BP) term of “anatomical structure morphogenesis” (p-value = 3.5e-07), “neuron projection morphogenesis”, “plasma membrane bounded cell projection morphogenesis” (p-value < 1e-5), and “mammary gland formation” (p-value < 2e-5), as well as other developmental process related terms, including “development growth” and “ureteric bud development” (p-value < 8e-5) (Fig. 3B**)**. These genes were also linked to critical biological processes related to “small GTPase mediated signal transduction” (p-value = 0.003), “glucosamine metabolic process” (p-value = 0.004), “glycoprotein biosynthetic process”, “glycoprotein metabolic process”, “protein kinase C-activating G protein-coupled receptor signaling pathway”, “insulin secretion”, “insulin-like growth factor receptor signaling pathway”. Furthermore, these genes were observed to be enriched in the “glycerophospholipid metabolism pathway” (p-value = 3e-3), “circadian entrainment” (p-value = 3e-3), “mesodermal commitment Pathway” (p-value = 2e-4), “endoderm differentiation” (p-value = 0.001) and “adipogenesis pathway” (p-value = 0.005) as well as other metabolic pathways^33^ (Fig. 3C).

### Comparisons with reported signals and genes associated with T1D complications

We investigated the overlap between our hDMCs and previously reported DMCs or genes associated with various T1D complications, particularly for coronary heart disease (CHD), Nephropathy, and Retinopathy, as well as metabolic memory. We first collected known CpG signals from six research or meta-analysis studies^14–17,34,35^. We did not observe high overlap rate when looking at hDMCs at p-value < 1e-3. However, if we relax the association p-value to nominal threshold of p-value < 0.05, we observed a few overlaps. Particularly, although none of the 18 DMCs that associated with incident CHD^34^ were identified as hDMCs in our study, we observed 4.2% (47 of 1119 overlapped CpGs) of CHD or other atherosclerosis-associated DMCs^35^. Among 23 DMRs to be associated with diabetic nephropathy risk in T1D patients^16^, three (13%) were showing hDMCs at the current study. Among 347 retinopathy associated DMCs^15^, 127 of them were tested in current study and 6.3% (8 of 127 CpGs) of them also showed hDMCs with p-value < 0.05. Regarding the metabolic memory study^21^, Chen et al. reported 403 and 379 DMCs associated with hyperglycemia in whole blood (WB) and monocytes (Mono), respectively. 185 and 173 CpGs were tested in this study and roughly 4–6% of them were also showing hDMCs at p-value < 0.05. In another recent study^18^, these authors reported 186 HbA1c associated DMCs through Illumina EPIC array. Four of the 75 CpGs tested in our study showed hDMCs at p-value < 0.05. Furthermore, if we further extend to the neighboring CpGs (i.e., considering CpGs within 500 bp distance of the reported DMCs), the overlapping rate increases. For instance, hDMCs (p-value < 0.05) were located at the neighbor CpGs of 7 (out of 18, 38.9%) and 462 (out of 1119, 41.3%) were CHD DMCs. Similarly, 48 of 127 (37.8%) retinopathy DMCs were identified as the neighbors of hDMCs within 500 bp distance. In addition, hDMCs were identified to be neighbors of 72 (out of 185, 38.9%) WB and 50 (out of 173, 28.9%) Mono DMCs associated with hyperglycemia as well as 43 (out of 75, 57.3%) HbA1c associated DMCs in Chen et al.’ studies (Additional file 4: Table S3).

## Discussion

Studying the longitudinal DNA methylation changes dynamic changes between two time points in the same patient is a novel approach that reduces the paramount difficulty of inter-individual methylation variations that arise when comparing different groups of patients. Within the observed years, all patients were exposed to various degrees of hyperglycemia, which was evaluated using the mean HbA1c value.

This study used the whole genome bisulfite sequencing (WGBS) technology to measure the DNA methylation of genome-wide 28 million of CpGs. Even if the CpG coverage or the number of measured CpGs was reduced due to the low sequencing depth of same samples, it still encompasses more than 10 million CpGs whereas Illumina array based 450 K or EPIC array assays covers only about 450 or 850 thousand CpGs respectively. Thus, the current WGBS data provided much more CpGs than previous array-based studies and offered the potential to detect novel hDMCs. Indeed, across the 8375, 815, and 78 hDMCs associated with HbA1c at p-value < 1e-3, 1e-4, and 1e-5, only ~ 3% were included in the EPIC array panel. Furthermore, only ~ 7–10% of these hDMCs were located within 100 bp distance to the EPIC array CpGs. Particularly, for the top 20 hDMCs listed in Table 2, only one of them showed neighboring CpGs in EPIC array with 100 bp distance, the 19 others would have gone undetected.

Interestingly, the loci harboring these DMCs were enriched in genes involved in “cell structure morphogenesis”, as well as other developmental process related terms, including “development growth”. These genes were also linked to critical molecular processes possibly involved in the pathogenesis of diabetic complications, such as “small GTPase mediated signal transduction”^36^, “glucosamine metabolic process”^37^, “glycoprotein biosynthetic and metabolic processes”^38^, “protein kinase C-activating G protein-coupled receptor signaling pathway”^39^, or “insulin-like growth factor receptor signaling pathway”^40^. When limited the enrichment analysis to hDMCs located within promoter or gene body regions, similar enriched terms were observed, as shown in Additional file 1: Figure S5. Particularly, biological processes related to “mesonephric development” and “developmental growth” were preserved. Additionally, pathway terms such as “Glycerophospholipid metabolism”, “Glycerolipid metabolism”, “Endoderm Differentiation” as well as “Mesodermal Commitment Pathway” were also retained.

Even more strikingly, several genes related to the 36 hDMCs associated with average HbA1c level showed a high degree of relevance for the mechanisms of T1D micro or macroangiopathy, some even concerning more specifically the retina and the renal glomeruli, the coronary arteries and the heart, tissues most concerned by long term complications of T1D^41^. We found that 13 genes out of 36 (Table 2) associated with hyperglycemia were involved in the regulation of angiogenesis, the vascularization and/or development of renal glomeruli and retina, the maintenance of coronary supply to the heart. For instance, the top signal was identified in the 14th exon of *BMPER* with nominal significance of p-value < 1e-7. BMPER is a secreted protein expressed by that modulates the function of endothelial cell precursors by binding directly to BMP2, BMP4, and BMP6, all important regulators of angiogenesis^42–44^. Another DMC identified with p-value 0.4e-6 is located nearby *BMP4*, a ligand of BMPER, involved in VEGF-dependent angiogenesis^45–47^. In endothelial cells, *BMPER* activates the fibroblast growth factors signaling pathway that is crucial in the promotion of angiogenesis and neovascularization^48^. Through its interaction with BMPs, *BMPER* seems to be involved in retinal angiogenesis and diabetic retinopathy^49–51^. Another DMC closely associated with hyperglycemia is *EBF1*, a transcription factor highly expressed in pericytes^52^, cells ubiquitously present in microvessels and interacting with endothelial cells to regulate angiogenesis and vessel stability^53^. *EBF1* is also expressed in podocytes, specialized pericyte-like cells covering the glomerular capillary endothelial cell layer of the Bowman’s capsule in the kidney^54^. Specialized renal pericytes known as mesangial cells provide physical support to glomerular capillaries, and may play an essential role in the development of diabetic nephropathy^55^. In addition, *EBF1* SNPs have shown a strong association with coronary disease^56^. Two other identified DMCs, *LYPD1* and *LMBR1* were also evidenced to play essential roles in angiogenesis^57,58^. *LYPD1* is involved in GATA6 modulation of angiogenesis and endothelial function^59^ and *LMBR1* also contributes to the regulation of angiogenesis^58,60–62^ Precisely, Shh signaling controls the expression of growth factors involved in neovascularization and vessel maturation and acts upstream of the most prominent angiogenic growth factor, VEGF^63,64^. In addition, *LMBR1* is essential for retinal vascular development^58^. *ROBO2* belongs to the 20 top genes associated with hyperglycemia-responsive DMCs, together with *SLIT2* (another hDMC associated gene at p-value < 1e-3). Slit-Robo signaling play important roles in regulation of sprouting angiogenesis^65,66^, notably for the glomerular and retinal vasculatures. In a glomerulus-enriched gene dataset, *ROBO2* expression was found to be down-regulated in diabetic nephropathy^67^. Glomerular capillary loops consist of interacting podocytes, endothelial and mesangial cells^68^. Robo receptors control glomerular angiogenesis by reducing endothelial cell proliferation and migration^69^. Disruption of the glomerular capillaries is seen in diabetic nephropathy and leads to proteinuria. Slit2-Robo2 signaling also affects the structural development of glomerular podocytes^70^. Slit2 signals via endothelial Robo2 to drive retinal neovascularization induced by VEGF-A^71^. *CRB2*, another hDMC associated gene in our top list, is predominantly expressed in retinal epithelium^72^ and kidney. *CRB2* contributes to hereditary focal segmental glomerulosclerosis^73^. Danio rerio injected with antisense morpholinos targeting crb2b showed abnormal glomeruli, with disorganization of the podocyte architecture^74^. *SIX2* plays an important role in regulating nephron development and connections between glomeruli and distal tubules^75–77^. *SIX2* also contributes to retinal development^78^. One of the hDMCs was found at the *BAIAP2L1* locus, also called *IRTKS*, expressed in primitive glomeruli^79^. Another identified hDMC is near the Thioredoxin-like protein 1 (*TXNL1)* gene. The thioredoxin system is an important regulator of postnatal angiogenesis via modulation of cellular redox status in endothelial cells^80^. *TXNL1* was found to be differentially regulated by diabetes in renal cortex proteome^81^. *TBC1D30* is another angiogenic modulator that influences VEGF and FGF2 action on angiogenesis^82^. *ARID5B* is a differentially methylated locus associated with diabetic nephropathy^83^.

Another set of 5 DMCs identified in the current study are located nearby genes or genomic sequences known to be associated with coronary disease, or heart failure a major cause of cardiovascular morbidity in T1D patients. The first is *PRELID2*, expressed in adult cardiomyocytes^56,84^. *MIATNB* is a long non-coding RNA associated with coronary disease^85,86^. miR4275 and *CCDC93* were also known to be associated with coronary disease^87,88^. Variants on *FAM241A* were associated with heart failure^89^.

Aside from genes possibly involved in vascular complications of T1D, we found a DMC nearby the *RNF157* gene known to be associated with cataract^90^, an interesting finding considering that hyperglycemia-induced osmotic damage to lens fibers might be the main mechanism of T1D associated cataract in young patients^91^.

We are not the first to study the relationship of methylation changes with chronic hyperglycemia with a longitudinal collection of samples. In a previous study, Chen et al. have collected blood samples 16–17 years apart and used Illumina 450–850 K arrays to compare a subset of 32 conventional T1D group members with a history of poor glycemic control and progression of microvascular complications with a subset of 31 former intensive group members with a history of good glycemic control and no progression of complications^18,21^. Differential methylation between the two groups persisted at several loci across the two time points, including a CpG site in the thioredoxin-interacting protein (*TXNIP*) gene. Miller et al. revealed that *TXNIP* DNA methylation is associated with glycemic control over 28 years in T1D^19^. These results provided strong evidence of a direct relationship between past glycemic exposure and DNA methylation in T1D. Furthermore, Soriano-Tárraga et al. identified that DNA methylation changes in *TXNIP* gene are also associated with sustained hyperglycemia in patients with type 2 diabetes mellitus^92^. Our observation complements these findings by studying the installation of dynamic methylation marks associated with early exposure to hyperglycemia, the period when metabolic memory is supposed to be imprinted in T1D patients to impact the risk of complications. It is interesting that similar to Chen’s observations^18^, we also identified couple of CpGs, located at the promoter and downstream of *TXNIP and with closest distances to the TSS of TXNIP*, showed reduced DNA methylation upon higher hyperglycemia exposure (*n* = 8 hDMCs with p-value < 0.05).

We also compared our findings with those from studies of DMCs associated with T1D complications, nephropathy^14,16^ retinopathy^15^, coronary heart disease^34^, or neuropathy^17^. These studies compared adult patients exhibiting complications with patients free of complications regardless of past exposure to hyperglycemia.

Due to the sequencing depth variability, we did not capture all the reported DMCs with sufficient sample coverages, roughly ranging from 28 to 49%. However, for those CpGs tested in our study, we found a few overlapping hDMCs at a replicate p-value of 0.05, particularly for the hyperglycemia exposure and retinopathy associated DMCs (4–6% of the reported DMCs). We further observed about 38–57% of these reported HbA1c or T1D complications associated CpGs showed significant hDMCs in our study when expanding up to 500 bp distance to them. This indicated that the potential biological relevance or replications of our discoveries.

Our study had strengths and limitations. It is the first WGBS based longitudinal follow up study in the same T1D patients covering the whole genome and containing much more CpGs than array-based studies. However, we acknowledge the relatively small sample set of this study and we did not have sufficient power to detect statistically significant associations at FDR q-value threshold of 0.05. We performed the hyperglycemia associated DNA methylation change analysis in easily available blood cells instead of cells such as endothelial cells, pericytes, podocytes, retinal cells, etc., in which vascular complications originate but are out of reach in clinical research. We did not have a replicate cohort to validate our findings, which would be our future work.

The current study used HbA1c measured periodically in the routine follow-up of T1D patients as a direct reflect of hyperglycemia. Consequently, our interpretation of the diabetic state was glucose-centric and focused on hyperglycemia as if it was the only metabolic driver of potential epigenetic changes. However, the perturbed metabolic state that characterizes a young T1D patient goes well beyond hyperglycemia. Increased lipids and ketone bodies, as well as multiple changes in circulating substrates or hormones, or tissue resistance to hormone action, occur in response to poor T1D control^93,94^ and are more or less correlated with HbA1c values. It is possible for example that some of the mechanisms that were discussed herein in the light of HbA1c and hyperglycemia were partially accounted for by dyslipidemia^95^ or insulin resistance^94^.

## Conclusions

In conclusion, exploring the relationship between DNA methylation and hyperglycemia in T1D patients uncovered critical molecular alterations associated with glycemic control and shed light on potential therapeutic targets. The identification of hyperglycemia-associated DMCs can serve as a basis for developing epigenetic biomarkers to help predict the risk of T1D complications long in advance. It is likely however that epigenetic marks of hyperglycemia are only part of the mechanisms involved in T1D induced micro- and macro-angiopathy.

## Electronic supplementary material

Below is the link to the electronic supplementary material.

Supplementary Material 1

Supplementary Material 2

Supplementary Material 3

Supplementary Material 4

## Data Availability

All the raw read files were submitted to European Genome-phenome Archive under the accession number EGAS50000000370.

## Abbreviations

BP: Biological process
CGI: CpG islands
CHD: Coronary heart disease
DHS: DNase I hypersensitive sites
DMC: Differentially methylated CpGs
DMR: Differentially methylated regions
GO: Gene Ontology
hDMCs: Hyperglycemia associated differentially methylated CpGs
KEGG: Kyoto Encyclopedia of Genes and Genomes
LM: Linear regression
Mono: Monocytes
PBMC: Peripheral blood mononuclear cells
SD: Standard deviation
TES: Transcription end site
TSS: Transcription start site
TXNL1: Thioredoxin-like protein 1
TXNIP: Thioredoxin-interacting protein
T1D: Type one diabetes
WB: Whole blood
WGBS: Whole genome-wide bisulfite sequencing
