# Supplementary material for: CpG methylation changes associated with hyperglycemia in type 1 diabetes occur at angiogenic glomerular and retinal gene loci
Source: Sci Rep. 2025 May 8;15:15999. doi: 10.1038/s41598-024-82698-9 (PMC12062505; doi:10.1038/s41598-024-82698-9)
Supplement: Supplementary file 4 — Supplementary Material 4 [file 41598_2024_82698_MOESM4_ESM.docx]

**CpG methylation changes associated with hyperglycemia in type 1 diabetes occur at angiogenic glomerular and retinal gene loci.**

**Xiaojian Shao^1,2^,** **Sophie Le Fur^3^, Warren Cheung^4^,** **Marie-Pierre Belot^3^, Kevin Perge^5^, Natacha Bouhours-Nouet^6^, Candace Bensignor^7^, Lucie Levaillant^6^, Bing Ge^8^, Tony Kwan^8^, Mark Lathrop^8^, Tomi Pastinen^4^, Pierre Bougnères^3^**

**Affiliations**

1 Digital Technologies Research Center, National Research Council Canada, Ottawa, ON, Canada, K1A 0R6

2 Department of Biochemistry, Microbiology and Immunology, University of Ottawa, Ottawa, ON, Canada, K1H 8M5

3 Groupe d’Études Diabète-Obésité-Croissance, Hôpital Bicêtre, 94276 Le Kremlin-Bicêtre,

4 Genomic Medicine Center, Children’s Mercy - Kansas City and Children’s Mercy Research Institute, Kansas City, MO 64108

5 Endocrinologie Pédiatrique, Hôpital Mère Enfant, Lyon, 69677 Bron

6 Département d'endocrinologie pédiatrique et de diabétologie, Hôpital universitaire d'Angers, 49933 Angers Cedex 9, France

7 CHU Dijon Bourgogne, Hôpital d’enfants, 21000 Dijon

8 Department of Human Genetics, McGill University and McGill Genome Center, Montreal, QC, Canada H3A 0G1

**Corresponding authors**

Xiaojian Shao [Xiaojian.Shao@nrc-cnrc.gc.ca](mailto:Xiaojian.Shao@nrc-cnrc.gc.ca)

Pierre Bougnères [pierre@bougneres.fr](mailto:pierre@bougneres.fr)

**Classification:** Biological Sciences - Medical Sciences


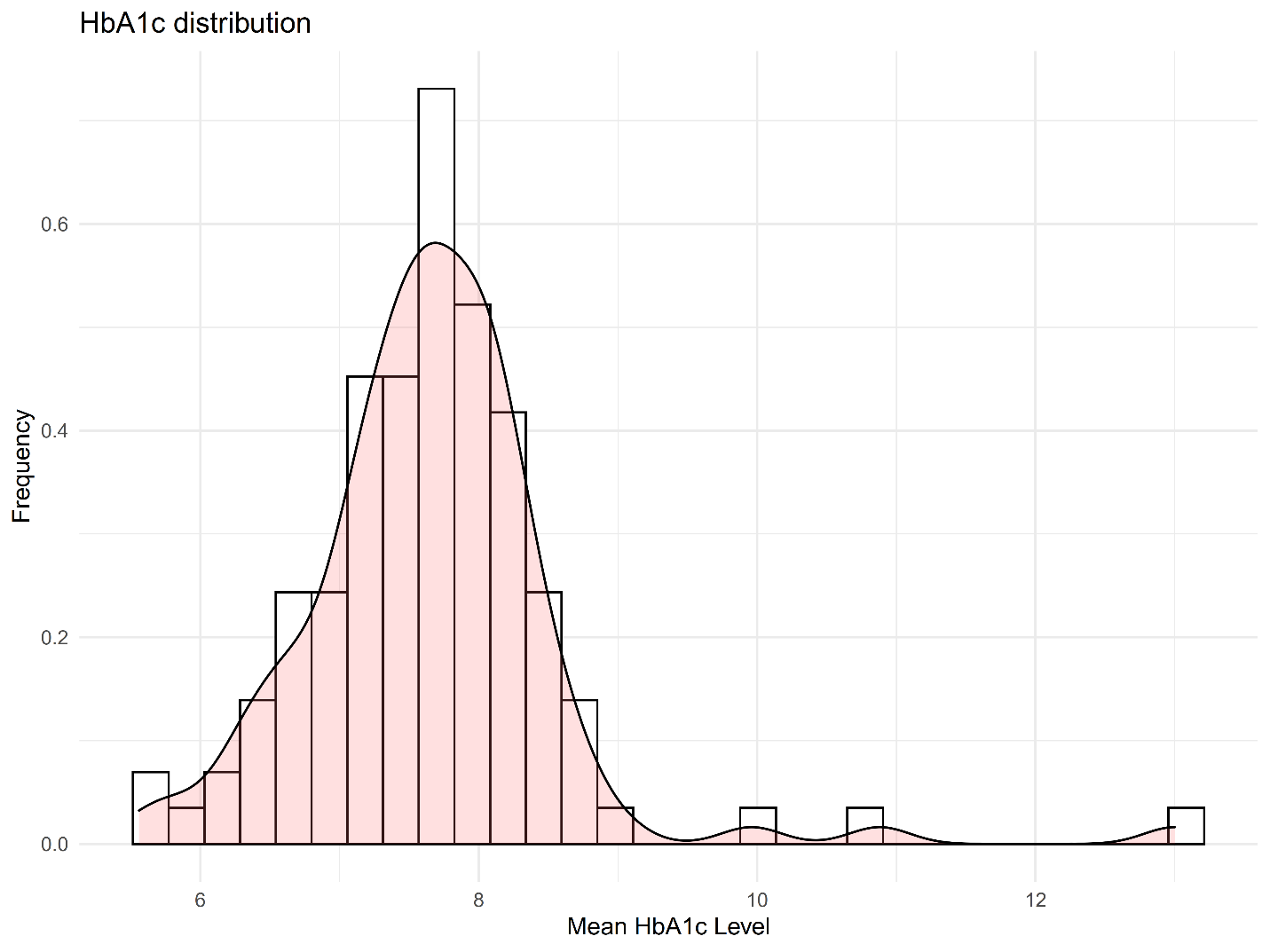


**Figure S1. Distribution of average HbA1c level in this study.** The average of mean HbA1c level is approximately 7.7 with a few patients having an average HbA1c level great than 10.


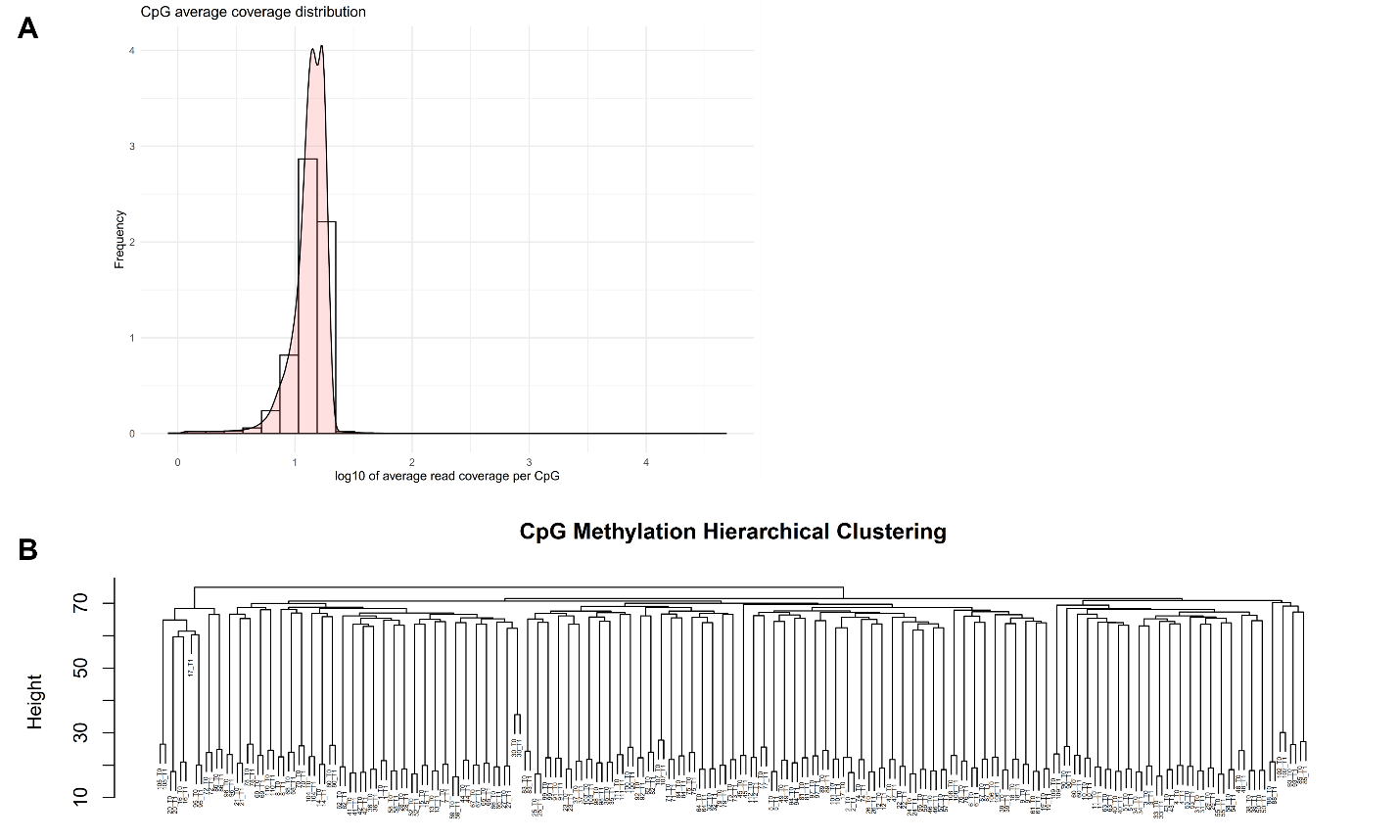


**Figure S2. Sequencing Statistics and Quality Control. (A). Distribution of mean read coverage of samples.** X-axis shows the log10 of read coverage with average read coverage of 15X. **(B). Hierarchical clustering based on top 1% variable CpGs in chr1.** DNA methylation profiles were used to perform the hierarchical clustering. Almost all the samples are well clustered with their paired longitudinal samples.


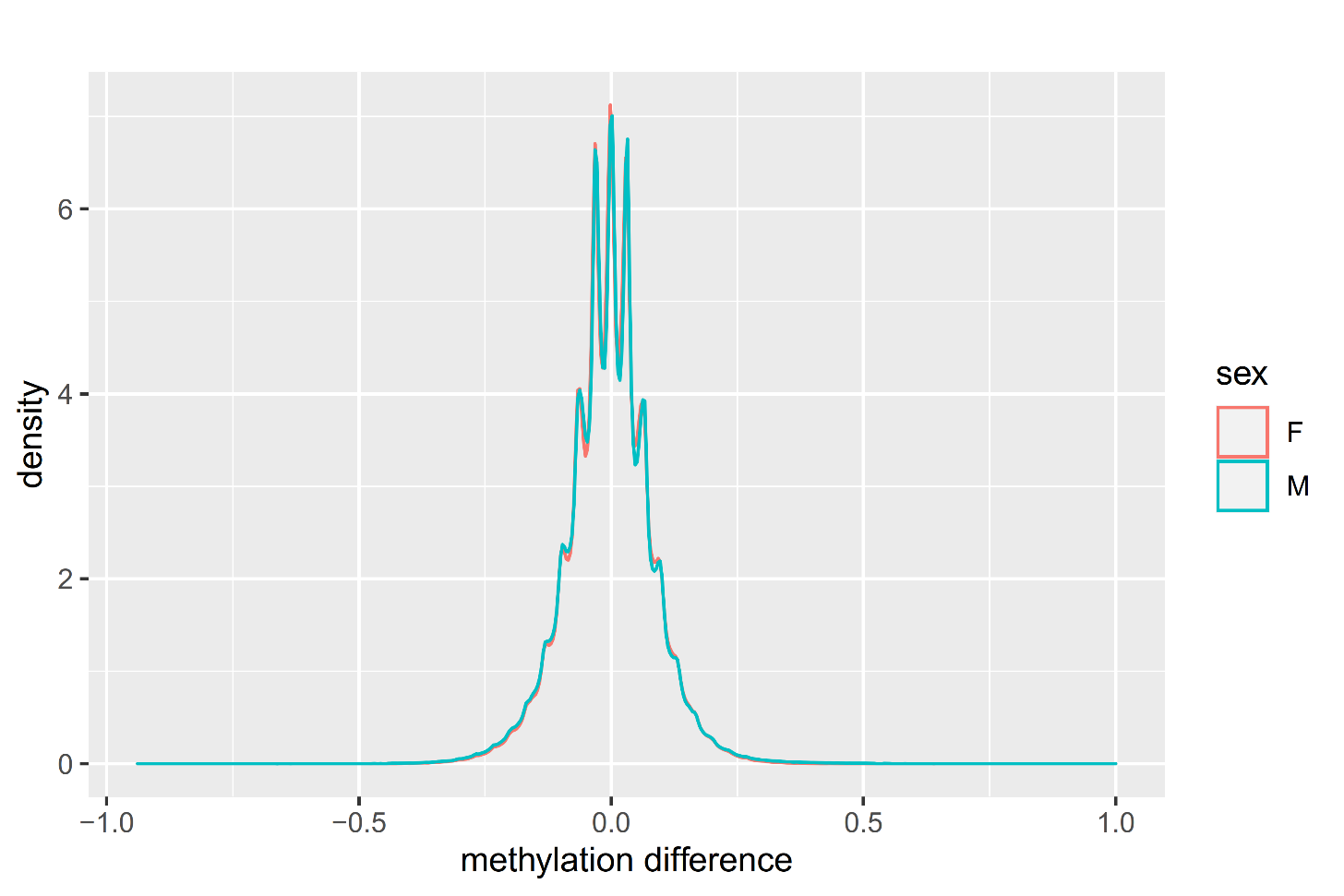


**Figure S3. Distribution of the DNA methylation changes between two time points for both male and female.** Similar pattens of DNA methylation difference were observed for both male and female patients.

**
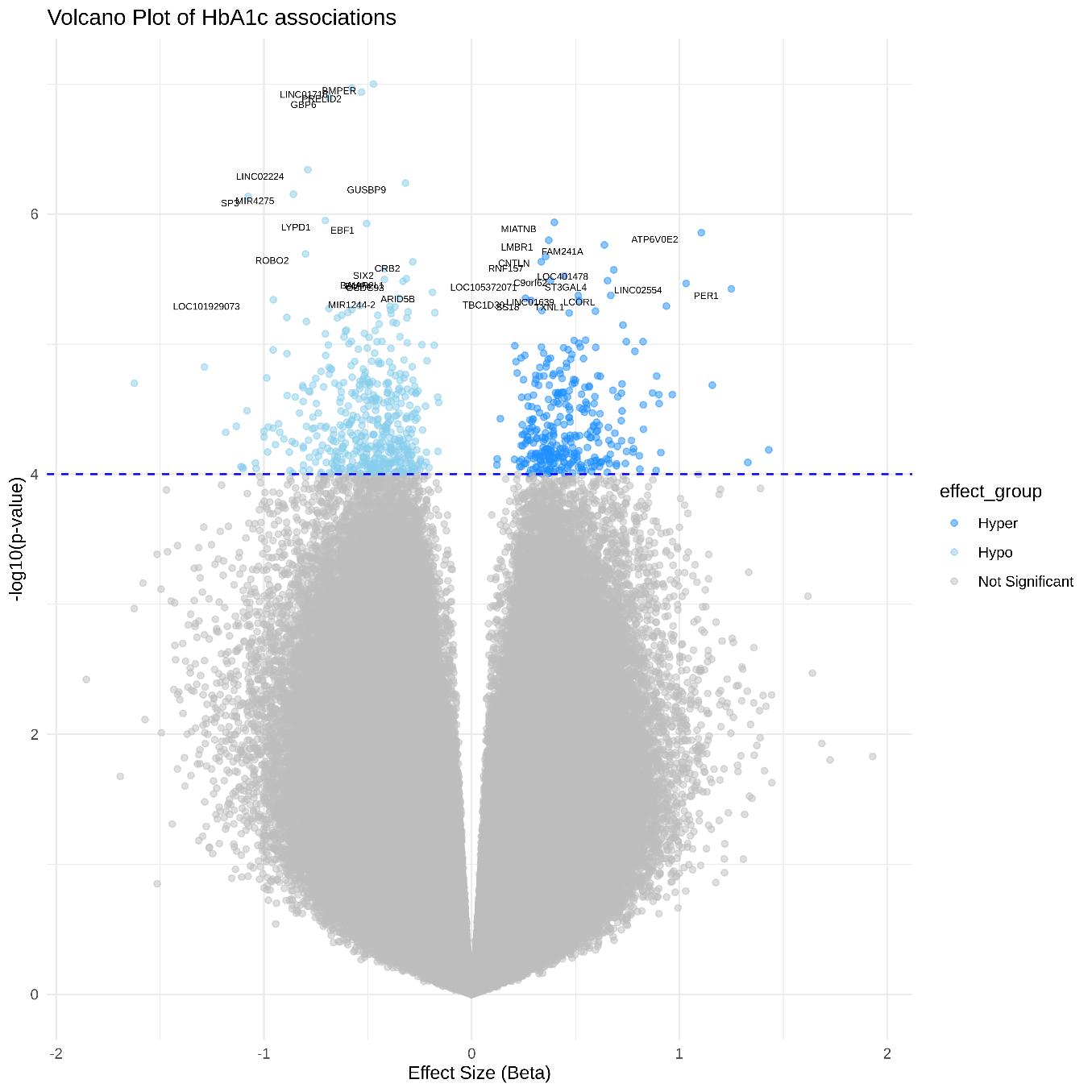
**

**Figure S4. Volcano plot for the HbA1c associations.** A suggestive p-value thresholds of 1e-4 is shown. Positive and negative associated signals were highlighted (i.e. hyper and hypo groups). The top hDMCs with p-value < 1e-5 were labelled, with associated genes or CpG coordinates displayed depends on whether the hDMCs were located at gene region or in intergenic regions.


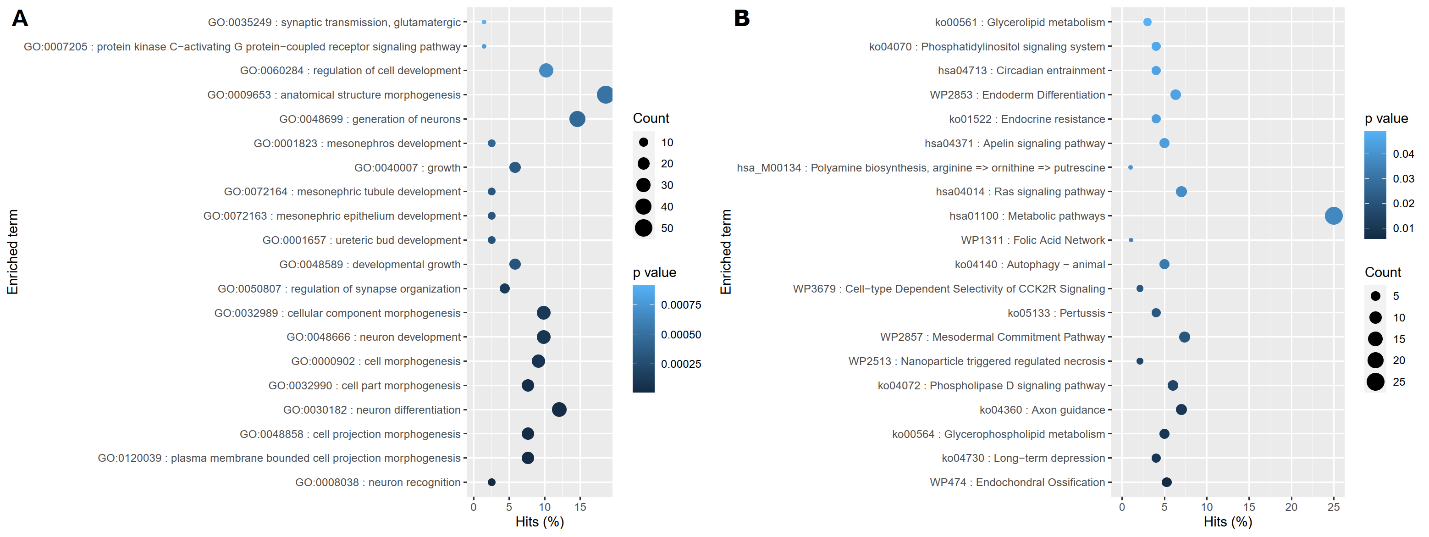


**Figure S5. Functional enrichment analysis of non-intergenic hDMCs.** Genes associated with non-intergenic hDMCs were used to perform the GO enrichment analysis. **(A)** GO BP enrichment results. **(B)** Wikipathway and KEGG pathway enrichment results. The enriched items were ordered by p-values.
